# Prevalence of *Helicobacter pylori* infection among children with primary nephrotic syndrome: a cross-sectional study

**DOI:** 10.4314/ahs.v20i4.15

**Published:** 2020-12

**Authors:** Ahmed Mahmoud, Ashraf Bakr, Afaf Elsaid, Yahya Wahba

**Affiliations:** 1 Mansoura University, Faculty of Medicine, Department of Pediatrics; 2 Mansoura University Children's Hospital, Biochemistry Section

**Keywords:** Children, *H.pylori*, primary nephrotic syndrome

## Abstract

**Background:**

Limited data are available about the prevalence of helicobacter pylori (*H.pylori*) infection among primary NS children.

**Objectives:**

To assess the frequency and risk factors of *H.pylori* infection among children with primary NS.

**Methods:**

A cross-sectional study was carried out in Mansoura University Children's Hospital, Egypt during the period from 2017 to 2019 including 100 NS children (NS group) and 100 healthy controls. NS group included 88 steroid sensitive (SSNS) and 12 steroid resistant (SRNS) cases. All patients were assessed for *H.pylori* infection using *H.pylori* stool antigen (HpSA) test. Statistical analysis was done using chi-square, fisher exact and Mann-Whitney tests.

**Results:**

With regard to HpSA test results, no significant differences were detected between control and NS groups (p = 0.193) and between SSNS and SRNS groups (p = 0.286). Concerning total biopsied cases and MCD (proven plus presumed) cases, no significant differences were found between those with positive and negative HpSA test (p = 0.648 and 0.126, respectively). The high dose of steroid therapy was associated with a higher risk of *H.pylori* infection among NS group (Odds ratio = 3.8; 95% confidence interval = 1.3–11.3).

**Conclusion:**

The current study negates the increased risk of *H.pylori* infection in children with primary NS.

## Introduction

Nephrotic syndrome (NS) is a relatively common pediatric disease^1^ with an immunological background proved by the successful therapeutic effects of steroids and immune-modulating drugs^2^,^3^.

*Helicobacter pylori* (*H.pylori*) are gram-negative bacteria that selectively colonize the gastric mucosa. The organism is spiral shaped with three to five polar flagella, and characterized by being urease, oxidase and catalase positive^4^. A recent meta-analysis revealed that about four billion persons were colonized with *H.pylori* worldwide in 2015^5^. In developing countries, *H.pylori* infection is highly prevalent and represents a great challenge^6^,^7^. In Egypt, this infection is very common reaching up to 72% among school children^8^.

Now, there is agreement that *H.pylori* is not only associated with gastric diseases but also with immune-mediated extra-gastrointestinal disorders^9^. It is associated with atopic dermatitis, Henoch-Schönlein purpura, idiopathic thrombocytopenic purpura, systemic sclerosis, recurrent aphthous stomatitis, alopecia areata and Sjogren's syndrome. In addition, *H.pylori* eradication could help in management of Behcet's disease. These effects could be attributed to the chronic systemic inflammatory state induced by the organism^10^,^11^.

Limited trials were conducted in the pediatric age group to detect the association between H.pylori infection and NS with contradictory results^9^,^12^,^13^. The current study assessed the frequency of *H.pylori* infection among children with primary NS and the related risk factors.

## Materials and methods

### Study design and participants

A cross-sectional comparative study was carried out from January 2017 to January 2019 including 100 primary NS children (NS group) and 100 healthy controls. NS patients were recruited from both inpatient wards and outpatient nephrology clinics of Mansoura University Children's Hospital, Egypt. NS group included 88 steroid sensitive (SSNS) and 12 steroid resistant (SRNS) cases. Among SSNS cases, 43 patients were infrequent relapsers, 30 patients were steroid dependent, 12 patients were first attack and 3 patients were frequent relapsers. The controls were recruited from children attending the same hospital with minor complaints e.g. mild gastroenteritis and pharyngitis.

Our study was accepted by Institutional Research Board of Medical Faculty of Mansoura University, Egypt (Code No: MS/16.08.35) and followed the Helsinki Declaration of 1975, as revised in 2000. Informed written consents were obtained from legal guardians of all study participants.

All primary NS children with persistent dyspeptic symptoms were included. NS was defined as the presence of edema, serum albumin < 2.5 gm/dl, nephrotic range proteinuria (≥40 mg/m^2^/h or protein/creatinine ratio >2 mg/mg) and elevated serum cholesterol and triglycerides levels^14^. Patients were labeled as SSNS if in remission, infrequent relapse, frequent relapse or steroid dependent. Steroid resistance means no remission despite daily prednisolone therapy at a dose of 2 mg/kg/d for four weeks. Patients were in remission if urinary albumin was trace or nil for three consecutive early morning specimens. Frequent relapse was defined as two or more relapses in the initial six months or more than three relapses in any 12 months of treatment. Steroid dependence means occurrence of two consecutive relapses within two weeks of steroid discontinuation or when on alternate day steroids^15^.

Patients were excluded if secondary NS, received *H.pylori* treatment within the last three months before participation in the study, received proton pump inhibitors within two weeks prior to sampling, or on an antibiotic treatment at the time of the testing procedure.

### Sample size

It was calculated using G*Power 3.0.10 program. Based on 65.6% prevalence among NS patients and 44.4% among controls (Moriyama et al., 2006)^16^, and assuming α (type I) error = 0.05 and β (type II) error = 0.2 and power = 80%; a sample size of 88 children was assumed for each group with an effect size = 0.425. We added 15% to overcome drop out, and the presumed sample size was 100 children in each group.

### History and clinical examination

Patients were subjected to detailed history taking with special emphasis on the response to steroids and the dose and duration of steroids therapy. Treatment of an initial episode of NS included oral prednisolone as a single daily dose starting at 2 mg/kg/d to a maximum 60 mg/d for 4 weeks followed by alternate-day dosing as a single daily dose starting at 1.5 mg/kg (maximum 40 mg/d) and continued for 2–5 months with gradual dose tapering. The steroid therapy should be given for at least 12 weeks^17^. Low-dose steroid was initiated for relapse of SSNS using oral prednisolone 1 mg/kg/d (maximum 40 mg/d) for a minimum of 7 days. Once the patient was in remission and/or had completed 7 days of steroid therapy, gradual tapering of prednisolone was done over a month. If the patient developed progressive edema or failed to get remission within 7 days of therapy initiation, the prednisolone dose was increased to the standard high-dose regime (2 mg/kg/d)^18^. All patients were assessed for gastritis symptoms as dyspepsia, vomiting, recurrent abdominal pain and epigastric tenderness.

### Laboratory evaluation

All participants were subjected to urine analysis, 24-h urine protein assessment/protein-creatinine ratio and serum levels of creatinine, albumin, cholesterol and triglycerides. Data about renal histopathology were retrieved from patients' files. Renal biopsy was done for only 38 cases (29 SSNS and 9 SRNS cases). Four SSNS cases (steroid dependent) and three SRNS cases were not biopsied due to patient guardians' refusal or current contraindications for renal biopsy (bleeding tendency).

*H.pylori* infection was assessed using *H.pylori* stool antigen test (HpSA test; ImmunoCard STAT! ®; Meridian Bioscience Europe, European Union; Catalog Number: 750220). HpSA test is simple, rapid, non-invasive, and can detect the active current infection. It is considered as a 2^nd^ generation rapid lateral flow immunoassay test using monoclonal anti-*H.pylori* antibodies. The specificity and sensitivity of the test are 99.2% and 94.3%, respectively as reported in the manufacturer's product information. The test value for *H.pylori* diagnosis was well recognized in previous reports^19^.

Stool samples were collected from all study participants, transported in airtight containers and tested as soon as possible but might have been held at 2°–8°C for 72 hours before testing. If testing could not be done within this time frame, specimens were frozen immediately upon receipt and stored at -20° to -80°C until tested. Each stool sample (5–6 ml) was mixed thoroughly with 1 ml diluent in a test tube. Then, we dipped the reaction strip in the test tube for 10 seconds, and read the results after 5 minutes. We considered the test negative if only a blue colored band (the control line) appeared across the white central area of the reaction strip, and positive if an additional pink red band (the test line) also appeared. Any pink red line, even very weak, was considered positive. The band intensity varied according to the concentration of the antigen in the specimen. Any color or line appearing after 10 minutes had no diagnostic value. The test was considered invalid if the control band was absent.

## Statistical analysis

SPSS version 21 was used to analyze data. Kolmogorov-Smirnov test was used to assess the data for normality. Qualitative data were described using numbers and percent. Chi-square and Fisher exact tests were used to describe the associations between the categorical variables. Non-parametric continuous variables were expressed as median (min-max), and analyzed using Mann-Whitney test. Logistic regression analysis was done for the risk factors associated with positive HpSA test among NS group. P ≤ 0.05 was considered significant.

## Results

Both NS and control groups were matched for the gender and age. The median age of NS group was 9 (1–17) years, while that of the control group was 8.5 (2–14) years (p = 0.083). Males constituted the majority of the study participants (67 children in NS group and 57 children in control group, p = 0.145).

With regard to HpSA test results, no significant difference was found between control and NS groups (Table 1, p = 0.193). Also, table 2 shows no significant difference between SSNS and SRNS subgroups (p = 0.286).

**Table 1:** Distribution of results of *helicobacter pylori* stool antigen test among children with primary nephrotic syndrome (NS) and control groups

HpSA test	NS group (n=100)	Control group (n=100)	p value
Positive	44 (44%)	35 (35%)	0.193
Negative	56 (56%)	65 (65%)	

HpSA: *Helicobacter pylori* stool antigen, NS: Nephrotic syndrome.

Data are shown as numbers and percent and analyzed by chi-square test.

**Table 2:** Distribution of results of *helicobacter pylori* stool antigen test among children with steroid sensitive NS and steroid resistant NS

HpSA test	Steroid sensitive NS (n=88)	Seroid resistant NS (n=12)	p value
Positive	37 (42%)	7 (58.3%)	0.286
Negative	51 (58%)	5 (41.7%)	

HpSA: *Helicobacter pylori* stool antigen, NS: Nephrotic syndrome.

Data are shown as numbers and percent and analyzed by chi-square test.

No significant difference was detected between total biopsied NS cases (n = 38) with positive HpSA test (20, 52.63%) and negative HpSA test (18, 47.37%) (p = 0.648). Mesangioproliferative glomerulonephritis and minimal change disease (MCD) were more frequent among biopsied NS group {16 patients (42.1%) and 10 patients (26.3%), respectively} followed by focal segmental glomerulosclerosis (5 patients, 13.2%), membranoproliferative glomerulonephritis (4 patients, 10.5%) and three patients (7.9%) had IgA nephropathy. MCD was the most frequent renal histopathology among biopsied NS patients with positive HpSA test (9 patients) with a significant difference when compared to those with negative HpSA test (1 patient) (p = 0.013). With regard to non-biopsied SSNS patients mostly presumed to be MCD (n = 59), 21 cases had positive HpSA test and 38 cases had negative HpSA test. Concerning the total MCD cases (proven plus presumed cases, n = 69), no significant difference was reported between those with positive HpSA test (n = 30; 43.48%) and negative HpSA test (n = 39; 56.52%) (p = 0.126) (Table 3).

**Table 3:** Distribution of renal histopathology among nephrotic syndrome children with positive and negative *helicobacter pylori* stool antigen (HpSA) tests

Renal histopathology	Positive HpSA test	Negative HpSA test	p value
Biopsied NS cases (total number =38)	20(52.63%)	18(47.37%)	0.648
-Proven MCD	9 (45%)	1 (5.55%)	0.013
-Mesangioproliferative GN	6 (30%)	10 (55.55%)	0.047
-FSGS	4 (20%)	1 (5.55%)	0.363
-Membranoproliferative GN	1 (5%)	3 (16.66%)	0.3
-IgA nephropathy	0	3 (16.66%)	-
Presumed MCD (non-biopsied SSNS, n=59)	21(35.59%)	38(64.41%)	0.0018
Total MCD (proven plus presumed MCD)	30(43.48%)	39(56.52%)	0.126

FSGS: Focal segmental glomerulosclerosis, GN: Glomerulonephritis, HpSA: *Helicobacter pylori* stool antigen, MCD: Minimal change disease, N: Number, SSNS: Steroid sensitive nephrotic syndrome. Data are shown as numbers and percent and analyzed by chi-square and fisher exact tests.

The high dose of steroid therapy was associated with a 3.8-fold higher risk of *H.pylori* infection among NS group (Odds ratio = 3.8; 95% confidence interval = 1.3–11.3) (Table 4).

**Table 4:** Logistic regression analysis of the risk factors associated with positive *helicobacter pylori* stool antigen test in children with primary nephrotic syndrome

Risk factors	Total	Positive HpSA test	p value	OR (95% CI)
Overall	100	44(44%)		
Age (years)				
≤ 6	31	15(48.4%)	0.554	1.3(0.6–3)
> 6	69	29(42%)		reference
Sex				
Male	67	27(40.3%)	0.288	0.63(0.27–1.5)
Female	33	17(51.5%)		reference
Total steroid duration (years)				
≤ 5	78	31(39.7%)	0.106	0.45(0.2–1.2)
> 5	22	13(59.1%)		reference
Steroid dose at time of study				
-Low dose (< 1mg/kg/d)	50	24(48%)	0.427	1.47(0.56–3.87)
-High dose (> 1mg/kg/d)	24	10(41.7%)	0.012	3.8 (1.3–11.3)
-Off steroids	26	10(38.5%)	-	reference

CI: Confidence interval, FSGS: Focal segmental glomerulosclerosis, GN:Glomerulonephritis, HpSA: *Helicobacter pylori* stool antigen, OR: Odds ratio. Regression model included all significant variables in bivariate analysis (Constant = -0.802, Model χ^2^ = 8.106; p = 0.044, predicted percentage = 63%).

## Discussion

Several studies were conducted to assess the possible role of *H.pyloi* infection in NS pathogenesis^16^,^19^–^21^. The current study reported an insignificant difference between healthy controls and primary NS children with regard to HpSA test. This observation was in contrast to Zajaczkowska et al^12^, Moriyama et al^16^ and Nagashima et al^20^ (Table 5). The only report that agrees with our results was that of Nutpho and Ukarapol^13^ (Table 5). This discrepancy could be explained by variability of the screening methods for *H.pylori*, different sample size and study design. This observation may negate the increased risk of *H.pylori* infection in children with primary NS.

**Table 5:** Various studies about *helicobacter pylori* infection and nephrotic syndrome

Study	Studied groups	Methods for *H. pylori* screening	Study outcome
Nagashima et al^20^	16 Adults with MN	Serology and immuno-staining for *H.pylori* in glomeruli	They found specific antigens in glomeruli and suggested *H.pylori* role in MN pathogenesis.
Zajaczkowska et al^12^	20 children with NS	Urea breath test	NS children were more prone to *H.pylori* than controls. The longer steroid therapy was associated with a higher *H.pylori* risk.
Moriyama et al^16^	32 Adults with primary MN & 243 controls	Serology	*H.pylori* was higher in MN than controls (p < 0.05). Its eradication reduced proteinuria in 3 of 4 MN cases who received steroids.
Nutpho & Ukarapol^13^	60 immunocompromised Thai children including 9 NS patients	Rapid HpSA test	One out of 9 NS children was positive for *H.pylori* antigens.
Sugimoto et al^21^	A 34-years old Japanese woman with stage I MN	Histopathology and immunostaining for *H.pylori* antigens in renal glomeruli	No *H.pylori* antigens deposited in the glomeruli. *H.pylori* eradication reduced proteinuria.
El-Latif et al^9^	60 immunocompromised Egyptian children including 15 NS patients & 30 healthy controls	Serology	Seroprevalence of *H.pylori* was higher in secondary immunodeficiency than controls (p = 0.02) and insignificantly higher in malignancy than NS (p = 0.51).
Caliskan et al^19^	59 adults (35 MN, 12 IgA nephropathy & 12 FSGS)	Rapid HpSA test & immunostaining for *H.pylori* antigens in renal glomeruli.	Negative staining for *H.pylori* antigens in renal glomeruli. *H.pylori* infection was higher in IgA nephropathy (83%) than MN (54%) (p = 0.045). *H.pylori* eradication decreased proteinuria in MN patients; however spontaneous remission could not be excluded.
Dede et al^24^	33 adults (10 IgA nephropathy, 7 MN, 6 mesangioproliferative GN, 5 FSGS and 5 membranoproliferative GN)	Urea breath test & histopathology	*H.pylori* eradication failed to improve proteinuria (p = 0.99).

FSGS: Focal segmental glomerulosclerosis, GN: Glomerulonephritis, HpSA: *Helicobacter pylori* stool antigen, MN: Membranous nephropathy, NS: Nephrotic syndrome

On reviewing the literature, some authors reported presence of *H.pylori* antigens in glomeruli of renal biopsy specimens, but failed to confirm the link between the organism and NS pathogenesis^20^. They depended upon the presumed hypothesis that *H.pylori* could increase the risk of NS through immune-mediated reactions induced by direct antigenic effects^20^ or auto-antibodies formation^22^,^23^. The effects of *H.pylori* eradication on the treatment response was also tested in few cohort studies. Some authors found insignificant improvement in proteinuria after *H.pylori* eradication^24^, while others failed to prove that the improvement in proteinuria was due to *H.pylori* treatment only and not due to spontaneous remission^19^. Even for studies that proved improvement in proteinuria due to eradication of H.pylori, the small sample size limits generalization of their results {three out of studied 32 cases in Moriyama et al^16^ and a case report by Sugimoto et al^21^}. Thus further randomized controlled trials (RCTs) with larger sample sizes are needed to provide more details about the exact pathogenic role of *H.pylori* in NS.

Moreover, the current study was the first to describe an insignificant difference between SSNS and SRNS children with regard to H.pylori infection. However, the high dose of steroid therapy was associated with a higher *H.pylori* risk among NS group, possibly due to the immuno-suppressant effect of the high-dose steroid 9.

In the current study, the age of patients was not considered a risk factor for *H.pylori* among NS group. This observation copes with a previous national report^9^. Other international studies with larger sample sizes proved that *H.pylori* infection rates increase with advancing age, mostly due to cumulative frequencies^25^–^27^. Also, the gender of patients was not a risk factor for *H.pylori* infection that agrees with many international reports^25^,^26^,^28^,^29^. However, other studies reported male predominance^27^,^30^. This discrepancy could be explained by differences in study design and sample size.

Unexpectedly, the total duration of steroids therapy did not increase the risk for *H.pylori*, possibly due to interrupted courses of therapy with variable intervals that increased the therapy duration among patients. This finding was in contrast to Zajaczkowska et al^12^.

Concerning total biopsied NS cases and MCD (proven plus presumed cases), the insignificant differences found between those with positive and negative HpSA tests suggest lack of association between *H.pylori* infection and renal histopathology in NS group. Our findings were in variance with Nagashima et al^20^

## Conclusion

The current study provides useful information about *H.pylori* prevalence among primary NS children in Egypt. Our study negates the increased risk of H.pylori infection in children with primary NS. The high dose of steroid therapy was associated with a higher *H.pylori* risk among those patients. Large scale RCTs multicenter studies are still needed.

## Limitations of the study

A single center association study with limited studied *H.pylori* risk factors, lack of long term follow-up and a potential selection bias of patients.

## List of abbreviations

CI: Confidence interval
FSGS: Focal segmental glomerulosclerosis
GN: Glomerulonephritis
H.pylori: Helicobacter pylori
HpSA: Helicobacter pylori stool antigen
MCD: Minimal change disease
MN: Membranous nephropathy
NS: Nephrotic syndrome
OR: Odds ratio
RCTs: Randomized controlled trials
SPSS: Statistical Package For The Social Sciences
SRNS: Steroid resistant nephrotic syndrome
SSNS: Steroid sensitive nephrotic syndrome
